# Promote Green Innovation in Manufacturing Enterprises in the Aspect of Government Subsidies in China

**DOI:** 10.3390/ijerph19137864

**Published:** 2022-06-27

**Authors:** Xiaojun Sun, Jing Tang, Shilong Li

**Affiliations:** 1 School of Management Science and Real Estate, Chongqing University, Chongqing 400044, China; sunxiaojun@cqu.edu.cn (X.S.); tangjing123@cqu.edu.cn (J.T.); 2 Center for Construction Economy and Management, Chongqing University, Chongqing 400044, China

**Keywords:** green innovation, government subsidies, public participatory environmental regulation, manufacturing enterprises

## Abstract

Green innovation is vital for manufacturing enterprises to achieve a balance between economic, environmental and social benefits. This paper empirically investigates the mechanisms of government subsidies, R&D investment and public participatory environmental regulation on green innovation in manufacturing enterprises, selecting a sample of 1308 manufacturing firms listed on Chinese A-shares from 2010–2019. The results show that government subsidies can significantly promote green innovation in manufacturing enterprises, with private enterprises being more pronounced. R&D investment plays a mediating role in green innovation in manufacturing enterprises, while public participatory environmental regulation has a negative impact. The moderating effect of public participatory environmental regulation on government subsidies is different on different green patents, with a more negative effect on green invention patents than on green utility model patents. Public participatory environmental regulation has a negative moderating effect on the green innovation of state-owned manufacturing enterprises while having no significant effect on private manufacturing enterprises.

## 1. Introduction

Under the triple pressures of demand contraction, supply shock and weakening expectations, achieving a comprehensive green transformation has become an urgent need for China’s sustainable economic development. As both the core creator of social wealth and the main claimant of natural resources, enterprises play an extremely important role in coordinating economic development and environmental protection [1,2]. Served as a tool for enterprises to achieve sustainable economic development, green innovation has become an essential tool for enterprises to maintain their core competitiveness [3,4]. In both developed countries such as German and Poland and developing countries such as Thailand and Hungary, green innovation drives resource efficiency and boosts financial performance [5,6,7]. Additionally, companies that introduce green innovation are also more likely to face lower financial risk exposure and to possess greater free cash flow than conventional firms [8,9]. In terms of China, the low-end manufacturing industry with high energy consumption and serious pollution accounts for a relatively high proportion of China’s manufacturing industry, which restricts the green transformation of the economy. As of 2020, the energy consumption per unit of product in China’s manufacturing industry is more than 20% higher than the international advanced level [10]. Facing the requirement of carbon peaking and carbon neutralization, promoting green innovation in manufacturing enterprises is of great significance to the low-carbon development of the manufacturing industry and the green transformation of the economy.

However, local firms have little incentive to undertake green innovation activities due to the externalities of environmental behavior [11,12,13]. On the one hand, local enterprises pay no cost for the extraction of natural resources; on the other hand, the benefits of environmental management and green innovation undertaken by local enterprises are shared by all economic parties, while the costs are borne alone [14]. Therefore, the key to incentivizing business participation in environmental governance is to internalize the problem of externalities, including charging companies for polluting and subsidizing them for cutting pollution [15,16]. In this context, government subsidies become a vital tool to inspire firms to undertake green innovation [17,18,19], as they can not only fill the funding gap in R&D directly but also reduce the uncertainty and risk of green innovation [20,21,22]. However, not unlike in developed countries, where people have voted to force the government to strictly enforce environmental laws and regulations [23], the phenomenon of “government failure” in green innovation of manufacturing enterprises is common in China due to incomplete information and weak supervision, which means the measures of the government are not always as effective as in theory [24,25,26]. As public concern for the living environment gradually increases, public participation in environmental governance has become an effective means of addressing market failure and government failure. It can regulate the environmentally unfriendly behavior and oversees green innovation actions of manufacturing enterprises by monitoring government enforcement and exerting public pressure [27]. With the gradual establishment of the tripartite system of “government-led, enterprise-dominated, public-participated”, exploring the impact of public participatory environmental regulation on green innovation in manufacturing enterprises has realistic significance.

Based on China’s market economy system, this paper empirically examines the impact of government subsidies and public participatory environmental regulation on green innovation in manufacturing enterprises, taking Chinese A-share listed companies as the research sample. The novelties of this study are as follows. First, the research theme is further focused on the impact of government subsidies and on general innovation to green innovation, and the empirical results at the firm level are more specific and practical. Second, by introducing public participation in environmental regulation as a moderating variable, it constructs a novelty perspective to investigate the impact of public participation as a regulatory force on green innovation in manufacturing firms. Finally, heterogeneity analysis in terms of green patents and ownership is further developed, which provides a theoretical basis for stimulating green innovation in different enterprises.

## 2. Literature Review and Hypotheses

### 2.1. Government Subsidies and Green Innovation

Though green innovation is the key link to the green and sustainable development of the manufacturing industry [28,29], considering the long period, uncertainty, and high transformation cost of green innovation [30], enterprises tend to invest in end-of-pipe treatment to obtain quick results when facing the pressure of environmental protection. Moreover, information asymmetry and moral hazard are prevalent between enterprises and investors as enterprises are wary of disclosing information about green innovation projects, which has led to higher financing costs and limited funding for R&D investment [31]. R&D risks are also a major impediment to corporate innovation. Technical risk, financial risk and personnel risk are concerned as the main risks that may appear in the R&D stage, including financing delays, changes of plans, failed experimental results and personnel changes [32,33,34]. Other than that, the commercialization risk of transferring R&D achievements into the mass production process must not be ignored. Manufacturing enterprises that engaged in green innovation activities need to invest large amounts of R&D capital and take huge risks, but it is impossible for them to reap the full benefits due to the positive externalities of green innovation, while other competitors can replicate or enjoy the company’s innovations at a lower cost [35]. As a result, the motivation for green innovation is reduced and some reasonable and effective green innovation projects are abandoned to avoid the R&D risks and “free-rider” behavior of other enterprises. Government subsidies can effectively compensate for the losses caused by the positive externalities of green innovation, alleviate the financial constraints of enterprises, and reduce the R&D risks of green innovation, thus becoming an important means for the government to incentivize manufacturing enterprises to carry out green innovation. First, government subsidies can help manufacturing enterprises overcome the constraint of limited internal resources and solve the problem of choosing between green innovation and end-of-pipe governance [36]. Moreover, government subsidies can reduce the marginal cost and disperse manufacturing enterprises’ R&D risks [7,37], thus promoting enterprises’ green innovation output. What is more, signal theory states that government subsidies are a sign of government support and trust in the project so that banks and entrepreneurs who originally had concerns about the green development of the enterprise willing to lend or invest in the enterprise [36]. In addition, the information asymmetry between manufacturing enterprises and external investors has led to external investors’ inability to have a comprehensive knowledge of the enterprises [38]. In such cases, government subsidies are regarded as a government-approved label that external investors are willing to trust and save their evaluation costs of the enterprises’ green development. Therefore, government subsidies can effectively alleviate the problem of insufficient funds for green innovation in manufacturing enterprises. Finally, to achieve higher environmental benefits, the government is more inclined to grant subsidies for projects such as clean energy and sustainable materials, thus guiding manufacturing enterprises to choose environmental directions for green innovation. Based on the above analysis, the first hypothesis is proposed.

**Hypothesis** **1** **(H1).** *Government subsidies can promote green innovation in manufacturing enterprises*.

### 2.2. The Mediating Effect of R&D Investment

Necessary R&D investment is not only the basic condition for manufacturing enterprises to carry out green innovation activities [39], but also has a considerable positive effect on green innovation output. Due to the high input and long cycle of green innovation, manufacturing enterprises have to constantly invest in human and material resources to maintain a satisfactory R&D environment for green innovation [40]. Government subsidies are considered to be an important source of funding with lower costs compared to operating income and loan investments. It can help to offset the risks arising from the innovation activities of manufacturing enterprises and enable them to increase R&D investment in green innovation [41,42,43,44]. Moreover, government subsidies can reveal the government’s support attitude, which is conducive to reducing the financing constraints of manufacturing enterprises. As a result, the ability of manufacturing enterprises to obtain funds is enhanced and more R&D investment can be put into green innovation [45,46]. Finally, government subsidies are monitored by government agencies, which provides a guarantee that enterprises will actually invest in green innovation activities after receiving funds. Based on the above analysis, the second hypothesis is proposed.

**Hypothesis** **2** **(H2).** *R&D investment plays a mediating role in government subsidies and green innovation in manufacturing enterprises*.

### 2.3. The Moderating Effects of Public Participatory Environmental Regulation

Government subsidies can solve the problem of low motivation for green innovation in manufacturing enterprises caused by the externalities of innovation to a certain extent. However, due to the limited resources, incomplete access to information and inadequate supervision of government regulatory departments, there are “government failures” phenomena in addition to the “market failure” phenomenon [47]. Pollution will continue if manufacturing enterprises believe their violations will not be detected by the government, and government subsidies that should have been used for green innovation are also at risk of being taken up by other activities, which can result in serious misplacement and waste of innovation resources [48]. When manufacturing companies keep causing environmental pollution that affects social welfare due to regulatory loopholes, the public will force manufacturing companies to reduce pollution emissions and improve environmental performance by reporting to government departments, boycotting their products or exerting public pressure [23,49]. As more and more public spontaneously supervise the pollution behavior of manufacturing enterprises, the regulatory force of public participatory environmental regulation has gradually formed. As a result, the environmental supervision that manufacturing enterprises are facing is more stringent. There are usually two options for manufacturing enterprises to reduce pollution emissions when it is compulsory. The first is to reduce pollution at the source by increasing investment in R&D and improving the green innovation capability of enterprises, so as to achieve a win–win situation between green development of manufacturing enterprises and ecological environmental protection. The second is to carry out pollution treatment, mainly focusing on end-of-pipe treatment [50]. Since China’s current public participation mechanism for environmental governance is still immature and the public lacks tolerance for enterprises’ pollution behavior, manufacturing enterprises tend to prefer the second approach in the context of increasing intensity of publicly participatory environmental regulation. Due to the lack of a strategic and holistic approach, the public often requires manufacturing enterprises to produce immediate environmental treatment effects when pollution is caused. Under such demands, insisting on investing in green innovation may expose manufacturing enterprises to negative public opinion and reduce their market value [51]. In addition, when the public feels dissatisfied with a company, they often express their dissatisfaction by boycotting its products and buying or supporting the products of the company’s competitors. End-of-pipe treatment can help manufacturing enterprises avoid this embarrassing situation, while green innovation has drawbacks such as long cycles and knowledge spillover compared to end-of-pipe treatments [52]. Considering the competitive pressure and business difficulties, it is reasonable for manufacturing enterprises to prefer choosing an end-of-pipe treatment to green innovation when the intensity of public participatory environmental regulation increases. However, this choice can crowd out R&D investment funds and reduce the innovation compensation effect of manufacturing enterprises. As a result, the level of green innovation in manufacturing firms decreases instead as public participation-based environmental regulation increases. Based on the analysis above, the following hypotheses are proposed in this paper.

**Hypothesis** **3** **(H3).** *Public participatory environmental regulation negatively moderates the relationship between R&D investment and green innovation in manufacturing enterprises*.

## 3. Research Design and Variable Description

### 3.1. Model Construction

Based on the above theoretical analysis, referring to the methods of Wen [53], Xu [54], and Pan [55], the following baseline regression model is established in order to test the effects of government subsidies, R&D investment and public participatory environmental regulation on green innovation in manufacturing enterprises.

For H1, to test the direct effect of government subsidies on green innovation in manufacturing enterprises, the benchmark model is established as shown in Equation (1) and set as Model 1.

(1) $$GI_{it}=\alpha_{1}GOV_{it}+\sum control_{it}+\mu_{1i}+\varepsilon_{it}$$

where *i* represents the enterprise; *t* represents time; *GI* represents green innovation; *GOV* represents government subsidies; *Control* represents control variables; *μ_i_* represents individual fixed effects and *ε_it_* represents a random disturbance term.

For H2, to investigate the mediating role of R&D investment in the path of government subsidies affecting green innovation in manufacturing enterprises, models 2 and 3 are constructed. If both the coefficients α_2_ and ρ_1_ are significant, then there is a mediating effect of R&D investment.

(2) $$RDI_{it}=\alpha_{2}GOV_{it}+\sum control_{it}+\mu_{2i}+\varepsilon_{it}$$

(3) $$GI_{it}=\alpha_{3}GOV_{it}+\rho_{1}RDI_{it}+\sum control_{it}+\mu_{3i}+\varepsilon_{it}$$

where *RDI* represents R&D investment.

For H3, the interaction term between public participatory environmental regulation and R&D investment is incorporated into model 3 to form model 4, to analyze whether the moderating effect of public participation-based environmental regulation exists. If the coefficient *θ*_1_ is significant, then there is a moderating effect of public participatory environmental regulation.

(4) $$GI_{it}=\alpha_{4}GOV_{it}+\rho_{2}RDI_{it}+\theta_{1}RDI_{it}\cdot PER_{it}+\sum control_{it}+\mu_{4i}+\varepsilon_{it}$$

where *PER* represents public participatory environmental regulation.

### 3.2. Variable Description

#### 3.2.1. Dependent Variable

Green innovation (GI): There are many ways to measure green innovation in existing studies, but they are mainly measured from industry or provincial perspectives, while those measured from corporate perspectives mostly use general innovation inputs and outputs as indicators, which hardly reflect environmental characteristics. This paper refers to previous scholars’ research and uses the number of green innovation patent applications as an indicator of green innovation [56,57,58].

#### 3.2.2. Independent Variable

Government subsidies (GOV): Government subsidies are measured by the ratio of government grants to the enterprise’s operating income, where government grants data are obtained from the financial statements of listed companies.

#### 3.2.3. Mediating Variable

R&D investment (RDI): Due to the large differences in size between enterprises, the ratio of R&D investment to enterprise operating income is used as the mediating variable in this paper.

#### 3.2.4. Moderating Variable

Public participatory environmental regulation (PER) is also known as voluntary environmental regulation or autonomous environmental regulation in academic circles. Due to the diversity of forms of public participation in environmental regulation, there is a wide variation in the quantification of this indicator, which is mainly expressed in the following ways: (1) The number of regional environmental pollution petitions [59]; (2) The number of complaints received by regional environmental protection departments [60]; (3) Total number of personnel of eco-environmental NGOs [61]; (4) The number of environmental protection proposals from the National People’s Congress [62]. From the perspective of continuity of data and representativeness of indicators, the ratio of the number of NPC recommendations to the total population of the region is selected as an indicator to measure the intensity of public participation-based environmental regulation.

#### 3.2.5. Controlled Variables

To exclude the possible interference of other factors on the influence of green innovation in manufacturing enterprises, with reference to other research on enterprise innovation [63,64,65,66], the following controlled variables are selected: (1) Enterprise size (SCA), measured by the logarithm of the enterprise’s total assets; (2) Ownership concentration (OC), measured by the shareholding ratio of the largest shareholder; (3) Current ratio (CR), measured by the ratio of current assets to current liabilities; (4) Cash flow ratio (CFR), measured by the ratio of current cash flow to current liabilities; (5) Return on equity (ROE), measured by the ratio of profit after tax to net assets; (6) Loan of asset ratio (LEV), measured by the ratio of total liabilities to total assets; (7) Operating income growth rate (OIG), measured by the ratio of growth in operating income for the current year to total operating income for the previous year; (8) Net profit (LNNP), measured by the logarithm of net income. The variable definition and description are shown in Table 1.

### 3.3. Data Sources and Statistical Characteristics

In 2009, China’s Ministry of Science and Technology issued the “National Industrial Technology Policy”, which clearly states that the government should increase financial support for enterprises to guide them to invest in R&D and enhance their innovation capabilities. In 2010, China became the world’s largest manufacturing country and has maintained its leadership since then. Until the outbreak of COVID-19 in December 2019, China’s manufacturing was hit severely and its purchasing managers’ index (PMI) started to fall in 2020. It is significant to explore whether government subsidies were effective in contributing to green innovation in manufacturing during the decade of 2010–2019. Therefore, this paper takes A-share listed manufacturing enterprises from 2010–2019 in China as the research object.

The patent data of listed enterprises are obtained from the Chinese Research Data Services (CNRDS), where the classification standard of green patents follows the green patent standards of the World Intellectual Property Office, State Intellectual Property Office of China, and Google Patent. The corresponding basic information and financial data of enterprises are obtained from the Wind database. Data on public participatory environmental regulation variables are mainly obtained from the China Environment Yearbook and the China Environment Statistical Yearbook for the years 2011–2020. The “Measures on Administrative Penalties for Environmental Protection” issued by the Ministry of Environmental Protection of China has come into effect on 1 March 2010. The “Measures on Administrative Penalties for Environmental Protection” issued by the former China Environmental Protection Administration was abolished at the same time. The “Measures on Environmental Administrative Penalties” are stricter in terms of penalty measures and environmental standards, therefore, 2010 was selected as the beginning year of the sample period. In late 2019, the large-scale outbreak of the COVID-19 epidemic led to substantial differences between the data after 2019 and the previous years, therefore, 2019 was selected as the end year of the sample period.

The specific screening steps for the sample enterprises in this paper are as follows: (1) Samples of special treatment (ST, *ST, SST) listed companies are excluded; (2) Samples delisted in the middle of the sample were excluded; (3) Data with missing values in variables are excluded; (4) Samples in Tibet, Hong Kong, Macao and Taiwan are excluded. This leads to a final sample of 1308 enterprises with an unbalanced panel of 9200 firm-year observations. In order to avoid interference caused by extreme outliers, all continuous variables are winsorized on the 1% and 99% quantile.

Table 2 shows the descriptive analysis of the main statistical variables. From the descriptive statistics, it can be seen that the minimum value of the green innovation index GI is 0 and the maximum value is 3.714. The gap between these two figures indicates a large disparity in the level of green innovation among manufacturing enterprises. The average value of 0.455 and the 50th percentile of 0 reveal that most manufacturing enterprises do not have green innovation output, indicating that the overall green innovation capability of manufacturing enterprises is not strong and needs to be improved.

## 4. Empirical Analysis and Robustness Tests

### 4.1. Analysis of Empirical Results

Considering the individual effects of the research subjects, this paper adopts a fixed-effects model to estimate the parameters through the Hausman test, and the estimation results are shown in Table 3. Model 1 explores the impact of government subsidies on green innovation performance, the regression coefficient of government subsidies is 0.104 and significant, indicating that government subsidies have a positive impact on green innovation in manufacturing enterprises. It is similar to the conclusion of Liu [22] and Huang [67] and confirms H1. The reason is that government subsidies can alleviate the constraint of insufficient innovation resources by injecting capital into the manufacturing companies, so the funding gap for green R&D projects is bridged [68,69]. Manufacturing companies will take the green innovation projects as the main target of R&D investment to obtain government subsidies, which further enhances the enthusiasm for green innovation and eventually leads to an increase in green innovation output.

Model 2 explores the relationship between government subsidies and R&D investment. The coefficient of government subsidies is positive and statistically significant at 1%, indicating that government subsidies have a positive effect on the R&D investment of manufacturing enterprises. Model 3 explores the relationship between government subsidies, R&D investment, and green innovation performance. The coefficient of government subsidies and R&D investment are both positive and statistically significant. Then, H2 can be verified from the regression results. This is because the entry of government subsidies can not only directly increase the cash flow of manufacturing enterprises, but also relieves the pressure of financing constraints of manufacturing enterprises by sending favorable signals to external investors. It indirectly improves the financing ability of manufacturing enterprises so that there will be more funds to invest in R&D activities. Based on the high risk and time-consuming characteristics of green innovation, the more funds are invested, the more abundant human and material resources can be provided, which is conducive to improving the effective output rate of green innovation as well as shortening the R&D cycle of green innovation.

Model 4 explores the moderating effect of public participatory environmental regulation in the process of R&D investment affecting green innovation in manufacturing enterprises. The coefficient of the interaction term between R&D investment and publicly participatory environmental regulation is negative and statistically significant at 5%, implying that H3 is correct. One possible reason for that is when pollution is detected, the public participatory environmental regulation requires the enterprises to take immediate actions and achieve rapid results [70]. Under such requirements, manufacturing enterprises have to invest in the re-cleaning of emissions, which leads to the extrusion of R&D funds for green innovation. As a result, the green innovation performance of manufacturing enterprises is reduced.

Conclusions about controlled variables can also be drawn from the regression results of Model 4. The coefficient of enterprise-scale is significantly positive, indicating that large-scale enterprises have advantages in green technology innovation. The existence of economies of scale in large-scale enterprises makes the average R&D cost decrease and R&D yield relatively high in the long run. The influence of the asset–liability ratio on the green innovation of manufacturing enterprises is significantly negative, indicating that the more liabilities of manufacturing enterprises, the lower the level of green innovation. Manufacturing enterprises lack sufficient funds to invest in green innovation R&D when the debt is high. The coefficient of operating income growth rate is significantly negative, indicating that the more the year-on-year growth of business revenue, the more it inhibits manufacturing enterprises from green innovation. This can be explained by the fact that successful business activities make enterprises more inclined to continue investing money in their operations to maintain a positive trend rather than to increase productivity through green innovation.

To avoid the error caused by multicollinearity among variables that affect the accuracy of empirical evidence, the VIF test is performed. The VIF test values of each model are less than 10, which means all variables passed the test, and there was no need to worry about multicollinearity.

### 4.2. Robustness Tests

To further ensure the robustness of the regression results, the number of green patents independently applied by the sample enterprises per year is used as the proxy variable for the level of corporate green innovation in this paper, denoted as GI1. The equations after the substitution of variables are shown below and are set as model 5, model 6, model 7 and model 8, respectively. The results are shown in Table 4.

(5) $$GI1_{it}=\alpha_{5}GOV_{it}+\sum control_{it}+\mu_{5i}+\varepsilon_{it}$$

(6) $$RDI_{it}=\alpha_{6}GOV_{it}+\sum control_{it}+\mu_{6i}+\varepsilon_{it}$$

(7) $$GI1_{it}=\alpha_{7}GOV_{it}+\rho_{3}RDI_{it}+\sum control_{it}+\mu_{7i}+\varepsilon_{it}$$

(8) $$GI1_{it}=\alpha_{8}GOV_{it}+\rho_{4}RDI_{it}+\theta_{2}RDI_{it}*PER_{it}+\sum control_{it}+\mu_{8i}+\varepsilon_{it}$$

Government subsidies significantly promote green innovation in manufacturing enterprises, R&D investment plays a mediating role in government subsidies promoting green innovation in manufacturing enterprises, and public participatory environmental regulation negatively regulates green innovation in manufacturing firms. The regression results of the core variables are the same as before, indicating the results of the research are robust and can be trusted.

## 5. Heterogeneity Analysis

### 5.1. Green Patents

Green patents can be divided into green invention patents and green utility model patents. Green invention patents are usually breakthrough innovations with high investment, high difficulty, and long return periods while green utility model patents often originate from low-cost imitation, with a small investment and a high probability of success [71]. Theoretically speaking, green invention patents have a more obvious effect on improving the economic performance and environmental benefits of enterprises. However, when manufacturing companies face financial difficulties, they prefer to develop green utility model patents. Is there a difference between the impact of green invention patents and green utility model patents? To answer this question, green patents are distinguished into green invention patents (GIP) and green utility model patents (GIU) for further heterogeneous discussion in this paper.

Columns (1) and (2) of Table 5 show the effects of government subsidies on manufacturing enterprises’ green innovation under different patent types. Government subsidies significantly improve the green innovation in manufacturing enterprises whether for green invention patents or green utility model patents, which is a further test of H1. The results of mediating effect of R&D investment are presented in columns (3) and (4) of Table 5. It can be seen that for green invention patents, R&D investment plays an incomplete mediating effect in the process of government subsidies affecting green innovation of manufacturing firms but a complete mediating effect for green utility models. Columns (5) and (6) of Table 5 show the regression results of the moderating effect of public participatory environmental regulation. It can be seen that public participation-based environmental regulation significantly inhibits the green innovation in manufacturing enterprises for both types of green patents, though the green invention patents are more affected. Compared with green utility model patents, a remarkable feature of green invention patents is the long R&D cycle. When the public is concerned about manufacturing enterprises’ green innovation, enterprises are expected to respond and improve quickly. Therefore, enterprises facing supervision pressure and public opinion pressure are more likely to carry out non-invention green patent innovation activities to quickly meet the need of the public. As a result, public participatory environmental regulation has a stronger inhibiting effect on the more difficult green invention patent innovation activities.

### 5.2. Ownership

China’s current basic economic system is based on public ownership and the joint development of multiple ownership systems. Under such a background, state-owned enterprises and private enterprises often show different characteristics in the development process. Therefore, it is necessary to consider the impact caused by different ownerships of manufacturing enterprises in examining the impact of different factors on green innovation in manufacturing enterprises. Manufacturing enterprises are distinguished into state-owned enterprises and private enterprises according to property rights in this paper, among which 438 are state-owned enterprises (SOE) and 930 are private enterprises (PE).

It can be seen from columns (1) and (2) of Table 6 that government subsidies can significantly promote green innovation in both state-owned enterprises and private enterprises, and this promotion is more significant for private enterprises. This can be attributed to the fact that SOEs generally adopt a more prudent strategy in operations due to their political functions. Under such a premise, SOEs might not necessarily invest the funds in green innovation with certain risks even when they received government subsidies [72]. In addition, since the management is usually appointed by the government, SOEs’ professionalism level is lower than that of private enterprises, which makes SOEs relatively inadequate in their ability to steer the direction of R&D innovation and have difficulty effectively transforming the government-subsidized resources into green innovation outputs. What is more, as SOEs are generally regulated by the government in terms of remuneration, the problem of managerial principal-agent is emerging which results in a lack of sufficient motivation to carry out green innovation projects. In contrast, private manufacturing enterprises have long been plagued by the problem of difficult and expensive financing due to financial mismatch. Government subsidies can effectively alleviate the financial difficulties of private enterprises, enabling them to spend more money on green innovation. Moreover, with less policy burden and more autonomy and flexibility, private enterprises can carry out green innovation activities more effectively. Private manufacturing enterprises also have the advantage of management ability, which is conducive to transforming resources into green innovation output.

From columns (3) and (4) of Table 6, it can be concluded that there is a significant mediating effect of R&D investment in SOEs but the effect is insignificant for private enterprises. Compared with non-state-owned enterprises, SOEs always receive more policy support and R&D subsidies from the government because of the natural connections between the government and SOEs. Government subsidies received by private enterprises are less and mostly directed to other purposes rather than R&D investment, so the mediating effect is not significant.

Columns (5) and (6) show that the negative moderating effect of publicly participatory environmental regulation on green innovation in SOEs is significant at 5%, while it is not significant for private manufacturing firms. SOEs’ business development is not profit-oriented and they need to take more social responsibility than private enterprises. Once there are environmental problems, SOEs will be the first target of public pressure since the public naturally demands more social responsibility from SOEs. In addition, under the influence of the government appointment system for SOE officials, many SOEs have multiple agents and lax supervision. In contrast, most of the markets in which private enterprises operate are close to perfectly competitive market structures. The fierce competition from existing competitors and potential entrants makes private enterprises cautiously comply with government regulations and advocate robust operation. In this case, even when the public is more concerned about environmental protection, it will not have a significant impact on private enterprises’ green innovation.

## 6. Conclusions

In recent years, green development has become the main theme of China’s economy. Based on the data of 1308 Chinese manufacturing A-share listed companies from 2010 to 2019, this paper explores the relationship between government subsidies, R&D investment, public participatory environmental regulation, and green innovation in manufacturing enterprises. The heterogeneity impacts are also discussed and tested by types of green patents and ownership. The main conclusions are as follows. First, government subsidies can significantly strengthen the incentive of manufacturing enterprises to engage in green innovation, which is more pronounced in private enterprises. Second, R&D investment plays a mediating role in green innovation in manufacturing enterprises while public participatory environmental regulation has a negative effect. Finally, the impact of public participatory environmental regulation is different on different green patents, with a more negative effect on green invention patents than on green utility model patents. Additionally, public participatory environmental regulation has a negative moderating effect on the green innovation of state-owned manufacturing enterprises while having no significant effect on private manufacturing enterprises. SOEs are not only profit-oriented but also have social responsibilities, the public is more willing to focus on SOEs and expects they will set an example when there are pollution problems [73,74]. Based on empirical results and conclusions, the following recommendations are proposed:

First, it is necessary to improve the government subsidy system and increase the strength of government subsidies. On the one hand, the government can adopt a more flexible subsidy system with a multi-pronged approach of R&D subsidies, environmental protection subsidies, tax incentives, and financial subsidies to motivate manufacturing enterprises to switch from ex-post pollution control to ex-ante green innovation. On other hand, the government should strengthen the supervision system of government subsidies, guarantee that government subsidies are put into practice, and maximize the effect of government subsidies.

Second, guiding and cultivating the public’s rational environmental awareness to make public participatory environmental regulation plays its full role. Public participation has become an important force in monitoring the pollution behavior of manufacturing enterprises. However, there may be a series of problems such as the “ NIMBY (not-in-my-backyard) syndrome “ and mass incidents if the public is not properly guided, which are not only detrimental to social stability but also to local economic development [75,76]. Therefore, the government should guide and cultivate the public’s rational awareness of environmental protection by strengthening the publicity of environmental knowledge, improving the public participation mechanism, and promoting the disclosure of environmental information, so that public participation can be effectively used.

Finally, the government should observe the management mechanism of state-owned manufacturing enterprises and increase the policy inclination toward private manufacturing enterprises. The government also needs to provide more subsidies to high-quality green innovation projects and guide innovation resources flow to private manufacturing enterprises with innovation capability. The reform of state-owned manufacturing enterprises should be continued so that government subsidies can help to promote their green innovation in the most efficient way.

There are also some limitations existing in this paper. There are still a large number of unlisted companies in the market, only using listed companies selected as a sample for the study will lead to a biased assessment of the actual situation. Combined with these limitations, future research on the green innovation of manufacturing enterprises can consider a longer time span, and this will incorporate the impact of the economic cycle into the empirical analysis. In addition, unlisted companies can also be included in the sample to obtain more comprehensive and realistic research results.

## Figures and Tables

**Table 1:** Variable definitions and descriptions.

Variable Type	Variable Name	Variable Code	Variable Description
Dependent Variable	Green Innovation	GI	The sum of the amount of green patents independently and jointly applied by enterprises this year plus one is taken as the logarithm.
Independent Variable	Government Subsidies	GOV	The ratio of government grants to the enterprise’s operating income
Mediating Variable	R&D Investment	RDI	The ratio of R&D investment to enterprise operating income
Moderating Variable	Public Participatory Environmental Regulation	PER	The ratio of the number of NPC recommendations to the total population of the region
Control Variables	Ownership Concentration	OC	The shareholding ratio of the largest shareholder
	Enterprise size	SCA	The logarithm of the enterprise’s total assets
	Net Profit	LNNP	The logarithm of net income
	Current Ratio	CR	The ratio of current assets to current liabilities
	Cash Flow Ratio	CFR	The ratio of current cash flow to current liabilities
	Loan of Asset Ratio	LEV	The ratio of total liabilities to total assets
	Return on Equity	ROE	The ratio of profit after tax to net assets
	Operating Income Growth Rate	OIG	The ratio of growth in operating income for the current year to total operating income for the previous year

**Table 2:** Descriptive statistics.

Variable	N	p25	Median	p75	Mean	Min	Max	Sd
GI	9200	0	0	0.693	0.455	0	3.714	0.843
GOV	9200	0.003	0.006	0.013	0.013	0	16.47	0.173
RDI	9200	1.510	3.280	4.620	3.627	0	151.6	3.855
PER	9200	0.037	0.049	0.071	0.058	0.006	0.217	0.034
RDI·PER	9200	0.063	0.154	0.275	0.212	0	13.67	0.288
CR	9200	1.140	1.678	2.838	2.569	0.361	18.90	2.807
SCA	9200	21.23	21.90	22.71	22.07	19.97	25.50	1.153
LEV	9200	0.242	0.387	0.545	0.400	0.050	0.907	0.197
ROE	9194	0.026	0.065	0.115	0.0550	−14.71	1.611	0.250
CFR	9200	0.010	0.046	0.0870	0.0480	−0.139	0.228	0.064
OIG	9200	−0.021	0.104	0.251	0.153	−0.453	2.077	0.336
OC	9200	0.378	0.633	1.080	0.896	0.108	4.915	0.835
LNNP	8278	17.73	18.63	19.62	18.68	15.05	22.55	1.469

**Table 3:** Regression results in direct, mediating and moderating effects.

Variables	(1)	(2)	(3)	(4)
	Model 1	Model 2	Model 3	Model 4
	GI	RDI	GI	GI
GOV	0.104 ***	4.669 ***	0.0577 ***	0.049 **
	(0.005)	(0.067)	(0.019)	(2.56)
RDI			0.010 **	0.021 ***
			(0.004)	(3.21)
PER				0.020
				(0.05)
RDI·PER				−0.188 **
				(−2.34)
SCA	0.187 ***	0.905 ***	0.178 ***	0.180 ***
	(0.024)	(0.119)	(0.024)	(7.52)
CR	−0.001	−0.008	−0.001	−0.001
	(0.003)	(0.029)	(0.003)	(−0.32)
ROE	0.0846	−1.292 *	0.097	0.094
	(0.163)	(0.662)	(0.163)	(0.57)
LEV	−0.192 **	−2.203 ***	−0.170 *	−0.170 *
	(0.089)	(0.387)	(0.089)	(−1.92)
LNNP	0.003	−0.121 *	0.004	0.003
	(0.013)	(0.064)	(0.013)	(0.20)
OIG	−0.071 ***	−0.810 ***	−0.063 ***	−0.063 ***
	(0.018)	(0.081)	(0.018)	(−3.49)
CFR	0.166	0.355	0.162	0.174
	(0.129)	(0.391)	(0.130)	(1.34)
OC	0.014	−0.079	0.015	0.009
	(0.020)	(0.051)	(0.020)	(0.44)
Constant	−3.656 ***	−13.02 ***	−3.528 ***	−3.544 ***
	(0.453)	(1.950)	(0.454)	(−7.84)
R-squared	0.032	0.258	0.033	0.034
Maximum VIF	4.66	4.66	4.66	5.09
Mean VIF	1.51	1.51	1.67	1.99

Notes: ***, **, and * indicate 1%, 5% and 10% of significance levels, respectively.

**Table 4:** Robustness test regression results.

Variables	(1)	(2)	(3)	(4)
	Model 5	Model 6	Model 7	Model 8
	GI1	RDI	GI1	GI1
GOV	0.064 ***	4.669 ***	0.036 ***	0.031 **
	(0.003)	(0.067)	(0.012)	(0.012)
RDI			0.00609 **	0.013 ***
			(0.002)	(0.004)
PER				0.013
				(0.248)
PER·RDI				−0.116 **
				(0.05)
SCA	0.116 ***	0.905 ***	0.110 ***	0.111 ***
	(0.015)	(0.119)	(0.015)	(0.015)
CR	−0.001	−0.008	−0.001	−0.001
	(0.002)	(0.029)	(0.002)	(0.002)
ROE	0.053	−1.292 *	0.060	0.058
	(0.101)	(0.662)	(0.101)	(0.101)
LEV	−0.119 **	−2.203 ***	−0.105 *	−0.106 *
	(0.055)	(0.387)	(0.0552)	(0.055)
LNNP	0.002	−0.121 *	0.003	0.002
	(0.008)	(0.064)	(0.008)	(0.008)
OIG	−0.044 ***	−0.810 ***	−0.039 ***	−0.039 ***
	(0.011)	(0.081)	(0.011)	(0.011)
CFR	0.103	0.355	0.101	0.108
	(0.080)	(0.391)	(0.080)	(0.080)
OC	0.009	−0.079	0.009	0.006
	(0.013)	(0.051)	(0.013)	(0.013)
Constant	−2.266 ***	−13.02 ***	−2.187 ***	−2.197 ***
	(0.280)	(1.950)	(0.282)	(0.280)
R-squared	0.032	0.258	0.033	0.034

Notes: ***, **, and * indicate 1%, 5% and 10% of significance levels, respectively.

**Table 5:** Regression results based on green patents heterogeneity.

Variables	(1)	(2)	(3)	(4)	(5)	(6)
	GIP	GIU	GIP	GIU	GIP	GIU
GOV	0.101 ***	0.034 ***	0.067 ***	0.001	0.0606 ***	−0.006
	(0.004)	(0.004)	(0.015)	(0.017)	(0.015)	(0.016)
RDI			0.007 **	0.007 **	0.016 ***	0.015 ***
			(0.003)	(0.003)	(0.005)	(0.006)
PER					0.143	−0.009
					(0.306)	(0.328)
PER·RDI					−0.157 **	−0.140 *
					(0.064)	(0.076)
Control	YES	YES	YES	YES	YES	YES
Constant	−3.036 ***	−2.057 ***	−2.943 ***	−1.963 ***	−2.958 ***	−1.975 ***
	(0.398)	(0.339)	(0.401)	(0.335)	(0.400)	(0.334)
R-squared	0.030	0.019	0.031	0.020	0.032	0.021

Notes: ***, **, and * indicate 1%, 5% and 10% of significance levels, respectively.

**Table 6:** Regression results based on heterogeneity of ownership.

	(1)	(2)	(3)	(4)	(5)	(6)
Variables	GI (SOE)	GI (PE)	GI (SOE)	GI (PE)	GI (SOE)	GI (PE)
GOV	0.901 *	0.102 ***	0.823 *	0.070 ***	0.785	0.0674 ***
	(0.492)	(0.005)	(0.492)	(0.022)	(0.477)	(0.023)
RDI			0.021 *	0.007	0.051 ***	0.010
			(0.011)	(0.005)	(0.018)	(0.007)
PER					1.405	−0.678
					(0.973)	(0.432)
PER·RDI					−0.648 **	−0.049
					(0.270)	(0.085)
Control	YES	YES	YES	YES	YES	YES
Constant	−5.838 ***	−3.113 ***	−5.465 ***	−3.038 ***	−5.667 ***	−3.030 ***
	(1.070)	(0.510)	(1.093)	(0.510)	(1.081)	(0.508)
R-squared	0.054	0.027	0.056	0.028	0.060	0.029

Notes: ***, **, and * indicate 1%, 5% and 10% of significance levels, respectively.

## Data Availability

The data presented in this study are available on request from the corresponding author. The data are not publicly available due to data management.
